# Helper T Cells in Idiopathic Membranous Nephropathy

**DOI:** 10.3389/fimmu.2021.665629

**Published:** 2021-05-20

**Authors:** Qihan Zhao, Haoran Dai, Xianli Liu, Hanxue Jiang, Wenbin Liu, Zhendong Feng, Na Zhang, Yu Gao, Zhaocheng Dong, Xiaoshan Zhou, Jieli Du, Naiqian Zhang, Hongliang Rui, Li Yuan, Baoli Liu

**Affiliations:** ^1^ Beijing Hospital of Traditional Chinese Medicine Affiliated to Capital Medical University, Beijing, China; ^2^ Shunyi Branch, Beijing Traditional Chinese Medicine Hospital, Beijing, China; ^3^ Beijing University of Chinese Medicine, Beijing, China; ^4^ Beijing Chinese Medicine Hospital Pinggu Hospital, Beijing, China; ^5^ Beijing Institute of Traditional Chinese Medicine, Beijing Hospital of Traditional Chinese Medicine Affiliated to Capital Medical University, Beijing, China; ^6^ Department of Nephrology, Affiliated Hospital of Nantong University, Nantong, China

**Keywords:** idiopathic membranous nephropathy (IMN), helper T cells (Th cells), autoimmune, antibodies, germinal center (GC)

## Abstract

Idiopathic membranous nephropathy (IMN) is an autoimmune disease in which the immune system produces an antibody response to its own antigens due to impaired immune tolerance. Although antibodies are derived from plasma cells differentiated by B cells, the T-B cells also contribute a lot to the immune system. In particular, the subsets of helper T (Th) cells, including the dominant subsets such as Th2, Th17, and follicular helper T (Tfh) cells and the inferior subsets such as regulatory T (Treg) cells, shape the immune imbalance of IMN and promote the incidence and development of autoimmune responses. After reviewing the physiological knowledge of various subpopulations of Th cells and combining the existing studies on Th cells in IMN, the role model of Th cells in IMN was explained in this review. Finally, the existing clinical treatment regimens for IMN were reviewed, and the importance of the therapy for Th cells was highlighted.

## Introduction

In 2009, Beck et al. (1) discovered the podocyte autoantigen, i.e., M-type receptor of secretory phospholipase A2 1 (PLA2R1), in the immune deposits of IMN, providing a key evidence of IMN as an autoimmune disease. Later, in addition to PLA2R, more IMN antigens were identified, including thrombospondin type-1 domain-containing 7A (THSD7A), neural epidermal growth factor-like 1 protein (Nell-1), and semaphorin 3B (sema3B), which were all self-components of podocytes (2). In recent years, the incidence of IMN has been increasing year by year, making it the most common primary glomerular disease (3). At present, it is widely accepted that the autoimmune reaction of antibodies and the circulation and combination of target antigens on the cell, formed *in situ* immune complex deposition in cells and basement membrane space, lead to cell destruction, basement membrane thickening, and glomerular filtration barrier damage, as well as proteinuria and low plasma protein concentration (4).

As a key component of the human adaptive immune system, Helper T (Th) cells play an auxiliary or regulatory role in the immune response by expressing CD4 (5). Before being stimulated by antigens and cytokines, CD4+T cells are in their initial state, namely, naive CD4+ T cells. Upon being stimulated, the naive T cells begin to differentiate into different lineages. The differentiation direction is influenced by T cell receptor (TCR) signaling and specific cytokines in the microenvironment, and the cell fate is determined by major activated transcription factors. At present, 5 subsets of Th cells are relatively well-defined: Th1, Th2, Th17, regulatory T (Treg) cells and follicular helper T (Tfh) cells (6).

Due to their importance to autoimmune response, possible roles of various subsets of Th cells in the induction, immune disorders, and antibody generation of IMN will be discussed, and new clinical therapeutic strategies will be presented.

## Understanding Helper T Cells

**Figure 1:**
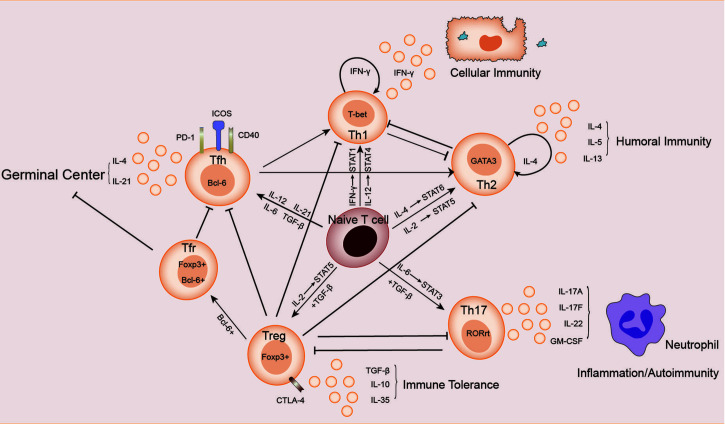
Relationship between Th cells subpopulations. Naive T cells differentiate in different directions under different conditions: IL-12 and IFN-γ activate STAT4 and STAT1 signaling, respectively, inducing the expression of the major transcription factor T-bet, and naive T cells differentiate in the direction of Th1 cells, which secrete cytokines such as IFN-γ and participate in cellular immunity. Th2 cells secrete cytokines such as IL-4, IL-5, and IL-13, which are involved in humoral immunity. In the presence of IL-6 and TGF-β, naive T cells differentiate towards Th17 cells, whose main transcription factor is RORγt. Th17 cells secrete cytokines such as IL-17A, IL-17F, IL-22 and GM-CSF, which are involved in autoimmune diseases or inflammatory responses. Treg cells secrete IL-10, IL-35 and TGF-β to maintain immune tolerance. The naive T cells differentiate towards Tfh cells in response to cytokines such as IL12, IL-21, IL-6 and TGF-β. Treg cells differentiate into Tfr cells in the germinal center. Tfh cells and Tfr cells together participate in the germinal center response.

The subsets of helper T cells are balanced and coordinated with each other, as shown in **Figure 1**. Th1 and Th2 subsets were the first ones discovered and explained by Mosmann et al. in 1986 (7). When the organism was infected with intracellular pathogens, such as viruses and bacteria, the naive T cells could be induced to differentiate into Th1 cells (8). Such differentiation is mainly promoted by IFN-γ and IL-12, which activate the major transcription factor T-bet through signaling transducer and activator of transcription (STAT)1 and STAT4 signaling, respectively, thus producing more IFN-γ in turn. IFN-γ is the major effector of Th1 cells functions to activate macrophage-mediated cellular immunity (6). IFN-γ also urges T-bet to produce a cascading amplification effect of Th1 cells through autocrine and positive feedback mechanisms (9). In contrast, Th2 cells mainly mediate humoral immune response and assist B cells to produce antibodies. IL-4 activates STAT6 signaling to promote the transformation of naive T cells to Th2 cells, which is regulated by GATA3, the major transcription factor of Th2 cells (10). IL-2 is also important for the formation of Th2 cells by activating STAT5 (11). IL-4 in Th2 cells also plays a similar role to the positive feedback mechanism of IFN-γ in Th1 cells, promoting Th2 cells differentiation (10). Th2 cells can also produce IL-5 and IL-13, etc., and participate in allergic reactions (6). There is an antagonistic relationship between Th1 and Th2 cells. First, when naive T cells receive antigen-presenting signals through TCR, a stronger TCR signal promotes Th1 differentiation, while a weaker TCR signal promotes Th2 differentiation (12). In addition, their major transcription factors T-bet and GATA3 are also inhibiting each other at both gene expression level and protein level (13, 14).

Approximately 10 years later after the discovery of Th1/Th2 cells, Sakaguchi et al. found a subpopulation (Treg cells) of CD4+T cells expressing the IL-2 receptor α (CD25) in mice that exacted immunosuppressive effects and maintained immune tolerance (15). Treg cells were derived from initial T cells induced by TGF-β alone and mainly regulated by the transcription factor forkhead box P3 (FoxP3) (16). Due to the grouping expression of CD25, Treg cells had a higher affinity with IL-2 than other Th subsets as it helped to achieve optimal inhibition of Treg cells through activation of STAT5 signaling (17). In general, Treg cells were found with high expression of CD4, simultaneous expression of CD25, Foxp3 (cytoplasm), and low expression of CD127 (IL-7 receptor α chain), constituting phenotype for such cells (18). Decrease in the number and/or function of Treg cells has been observed in patients with a variety of autoimmune diseases and mouse models (19). Treg cells have been identified with functional plasticity and different transcriptional characteristics in response to different types of immune responses and environments, thus playing a greater role of immunosuppression. There is also a subset of follicular regulatory T cells (Tfr) located in the germinal center (GC) that, in addition to expressing Foxp3, also express the chemokine CXCR5 and transcription factor Bcl-6, which are also markers of Tfh cells (20). The function of Tfr cells is to inhibit GC reaction and plasma cell differentiation, which is in balance with Tfh cells.

Until 2005, a new subset of Th cells known as Th17 cells which can secrete IL-17 to regulate tissue inflammation was discovered (21, 22). The development of Th17 cells rely on both the induction of TGF-β and the action of the inflammatory factor IL-6. They activate the major transcription factor RORγ-T through the STAT3 signaling pathway, which determines the differentiation of naive T cells to Th17 cells. This induction of IL-6 can also be enhanced in the presence of other cytokines, including IL-1β, TNF-α, IL-23, and IL-21 (23–25). Th17 cells produce IL-17A, IL-17F, IL-22 and granulocyte-macrophage colony-stimulating factor (GM-CSF), recruit inflammatory cells such as neutrophils, and promote inflammation at the infected site (26). An increase in Th17 cells has been observed in a variety of forms of autoimmune diseases, including inflammatory bowel disease (IBD), psoriasis, rheumatoid arthritis (RA), etc. (26), which is contrary to the observed reduction or suppression of Treg cells. There is a balance between Th17 and Treg cells: first, they compete for TGF-β at the site of differentiation; second, both STAT5 and Foxp3 in Treg can inhibit Th17 differentiation, while STAT3 signaling in Th17 can down-regulate Foxp3. All these lead to the differentiation of naive T cells in two different directions under different conditions. It was much believed that the imbalance of Th17/Treg cells was the key to the pathogenesis and therapeutic target of autoimmune diseases (27, 28). Yet, the cause for such imbalance still remains unknown.

**Figure 2:**
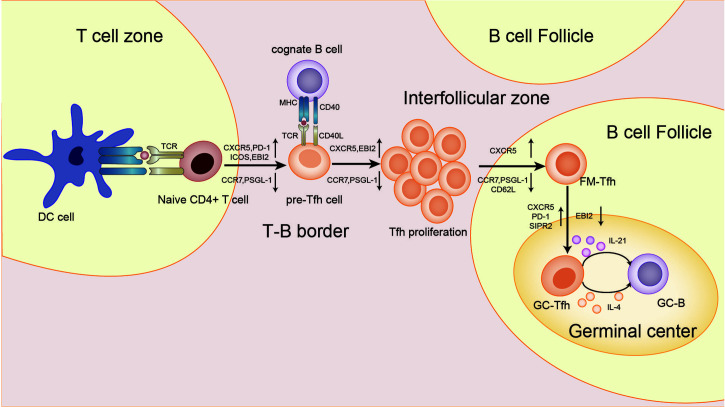
Differentiation and development of Tfh cells. First, in the T cell zone, the naive T cells receive the antigen presentation signal from the DC cells, and Tfh cells begins to differentiate. T cells expressed CXCR5, PD-1, ICOS, and epstein-barr virus-induced gene 2(EBI2), while CCR7 and P-selectionglycoproteinligand-1(PSGL-1) were down-regulated to obtain the pre-Tfh cell phenotype. At the T-B border, cognate B cells interact with T cells to maintain the Tfh cell phenotype. After that, the T-B cell complexes move from the border to the interfollicular zone, where more proliferation takes place. Next, Tfh cells are about to enter the follicle, and the signal from the bystander B cells further upregulates CXCR5 and suppresses CCR7, PSGL-1, and CD62L. Finally, Tfh cells in the follicular fimbria up-regulated CXCR5, PD-1, and sphingosine-1-phosphate receptor 2(S1RP2) surface molecules, down-regulated EBI2, and became GC-Tfh cells. The expression of IL-21 and IL-4 by GC Tfh is essential for the survival, proliferation and differentiation of germinal center B cells.

Several groups of studies have identified a type of CXCR5+Th cells that have a specific and preferred helper function to B cells in follicles (29–31), known as follicular helper T(Tfh) cells. The main transcription factor of Tfh cells is Bcl-6, which is essential to Tfh formation, assistance to B cells and GC formation (32–34). The expression of Bcl-6 inhibits differentiation of CD4+T cells in directions other than Tfh cells (33), and also hinders the expression of Th1, Th2, Th17 and Treg-related functional receptors (35, 36). In humans, IL-12, IL-21, IL-6, IL-23, and TGF-β synergistically promote Tfh cells, but TGF-β inhibits Tfh cells development in mice (37–40). IL-2 inhibits STAT3 and Bcl-6 by phosphorylating STAT5, and upregulates Blimp-1, thereby inhibiting Tfh cells (41, 42). Tfh cells also secrete IL-21 and express surface molecules programmed cell death protein 1 (PD-1) and recombinant Inducible T cell co-stimulator (ICOS) (43–45), which are critical for regulating the development, migration and function of Tfh cells. Differentiation and development of Tfh cells is mainly accomplished in secondary lymphoid organizations (SLOs). Through the interaction with B cells, Tfh cells gradually migrate from the T cell zone, through the T-B border, to the B cell follicles and germinal center, and finally form GC Tfh cells (46–49), as shown in **Figure 2**. GC Tfh cells are necessary to maintain GC response and cause three outcomes of B cells: A, differentiation into long-term memory B cells, waiting to be exposed to antigen again; B, differentiated into long-lived plasma cells to continue to produce antibodies; C, re-entry into the dark zone for more proliferation and somatic hypermutation (50–52). Owing to its heterogeneity and plasticity, GC Tfh cells are also able to adapt to different types of immune responses. In addition to secreting IL-21, Tfh cells can also produce IL-4 in response to Th2-mediated antibody response (53).

Since it is difficult to obtain SLOs from patients, attention has been paid to circulating cells with a Tfh phenotype. Some CD4+T cells in the blood with a Tfh-like phenotype (CXCR5+) subpopulation, but without Bcl-6 expression, are referred to as circulating Tfh (cTfh) cells (52). Although the relationship between cTfh cells and true Tfh cells in SLOs is unclear, the frequency of cTfh and its subsets are associated with influenza vaccines, chronic infections, and autoimmune diseases (54–58). Therefore, circulating CXCR5+CD4+T cells are currently considered to be the circulating responders of Tfh cells. According to the different expressions of CXCR3 and CCR6, cTfh can be divided into three subsets expressing different cytokines: A, CXCR3+CCR6-cTfh1, which can secrete IFN-γ; B, CXCR3-CCR6-cTfh2, which can secrete IL-4, IL-5 and IL-13; C, CXCR3-CCR6+cTfh17, which can secrete IL-17A, IL-17F and IL-22 (59). In addition, the activation status of cTfh cells can be distinguished according to the expression of ICOS and PD-1 cTfh2 and cTfh17 can secrete IL-21, which can effectively induce proliferation and differentiation of juvenile B cells and antibody class conversion (59–62).

## Helper T Cells in IMN

### Th Cells and Induction of IMN

There are many inducing factors of autoimmune diseases, such as the change in autoantigen, the abnormality of immune system, genetic factors, gender and age, etc., as well as their combined forces (63). IMN is usually caused by a single antigen, of which PLA2R accounts for 75%, and 10%-20% of IMN patients have not yet been identified with their antigens (64). Exposure to autoantigen is the major incidence reason, and no direct evidence has been found to reveal this process in IMN. Considering PLA2R as an example, anti-PLA2R antibodies in serum of IMN patients can bind to PLA2R antigen *in vitro* in a non-reduced state (65), which suggests that the antibody-bound epitopes require PLA2R spatial epitopes and are maintained by disulfide bonds (66). In China, the incidence of IMN is positively correlated with air pollution reflected by PM2.5 (67). We and Paul Brenchley et al. have proposed the hypothesis that lung tissue is stimulated by PM2.5 to cause an inflammatory environment, leading to exposure of PLA2R1 pathogenic epitopes in a strong oxidative microenvironment and then inducing the pathogenesis of IMN (68, 69). Recently, several studies have indeed found enhanced expression of Th17 cells and up-regulation of IL-17 and other cytokines in IMN, suggesting that there is indeed an inflammatory environment in IMN (70–72). Why has PLA2R become the main autoantigen of IMN? This may be related to genetic predisposition. At present, HLA-DQA1 and PLA2R allele risk loci have been found in IMN, which can promote the delivery of antigen epitopes to T cells through major histocompatibility complex (MHC) class II (73, 74), and CD4+T cells receive antigen signals through TCR.

In addition to the exposure of epitopes, the pathogenesis of IMN also involves the breakdown of autoimmune tolerance, including central and peripheral immune tolerance. The production of autoreactive T cells and B cells matters a lot, and the question is how they can escape the numerous tolerance checkpoints. In the process of thymus development of T cells, the V region gene of TCR is rearranged (75). This process may produce TCR against autoantigen, which can be eliminated by negative selection. However, this process may be abnormal in autoimmune diseases, causing abnormalities in the TCR library of T cells arriving at the periphery. Not long ago, Yu Zhang et al. (76) used T-cell receptor repertoire high-throughput sequencing (TCR-HTS) to analyze the TCR β chain repertoire of the circulating T lymphocytes of IMN patients. The result showed that IMN had lower diversity of VJ cassette combination in peripheral blood and a decrease in TCR lineage diversity. A decrease in TCR diversity of peripheral T cells has also been observed in patients with compulsive spondylitis and systemic lupus erythematosus (77, 78). This may explain why autoreactive T cells have escaped central immune tolerance, or why TCR has a shared sequence in patients, increasing the risk of autoimmune diseases (79). Peripheral immune tolerance may also play a key role in autoimmune diseases (80). Treg cells are the key to maintaining peripheral immune tolerance. A large amount of evidence shows that IMN has a reduced proportion of Treg cells in serum and decreased expression of Foxp3 (72), as well as impaired activation and inhibition of Treg cells (81). However, the expression of Treg cells in patients improved by rituximab treatment was significantly up-regulated, and the proportion of Treg cells had a prognostic effect on the treatment of rituximab (82, 83).

Immune response of IMN is dominated by humoral immunity (84), during which differentiation and development of autoreactive B cells are crucial, and Tfh cells play the role of peripheral immune tolerance checkpoint (85, 86). The B cell pool of healthy adults contains a large number of autoreactive B cells, but they have a low affinity and therefore do not cause disease (87, 88). Autoimmune diseases, including IMN, require high affinity with disease-causing antibodies (89, 90), suggesting that these plasma cells that produce these antibodies have undergone affinity maturation and somatic hypermutation (SHM) in GC. In fact, most GC-B cells experience apoptosis, and only a small portion survives and differentiates into memory B cells or plasma cells to leave GC (91–93). GC-B cells can survive and develop only with the assistance of T cells. A competitive model was first proposed, which was positively correlated with B cell receptor (BCR) affinity and antigen presenting ability (52). Professor Carola G. Vinuesa later described this competitive mechanism as positive selection and negative selection of Tfh cells (85, 94). Positive selection meant that Tfh cells provide survival signals to GC-B cells through CD40L, IL-4, IL-21 and other cytokines (94). Negative selection referred to the process Tfh cells transmit death signal to GC-B cells *via* CD95L. Mice with CD95L deficiency would develop autoimmune diseases (94). B cells without CD40L signal went to apoptosis (95), and the homologous interaction of T-B cells could make B cells enter the dark area again for further division and SHM (85). Studies have shown that the reduction of SHM is associated with the impairment of B cell tolerance, and the increase of cTfh cells and IL-21 in such patients (86). Restrictions on the number and quality (secreting cytokines) of Tfh cells create an environment in which GC B cells must compete for help, making it difficult for some low-affinity B cells, such as autoreactive B cells, to proliferate and differentiate. However, when the amount of Tfh cells increase abnormally, this checkpoint will be damaged, and the loosening of the floodgate will allow some autoreactive B cells to proliferate and differentiate, producing antibodies, and leading to autoimmune disease, which has been confirmed in Sanroque mice (45, 96, 97). In addition, Tfh cells were associated with the occurrence of autoimmune responses in chronic inflammation (98) as well as the process of antigen simulation (99), which has not been further investigated yet.

In fact, no matter the central or peripheral immune tolerance is abnormal or not, the resulting diseases are often multi-antigen pathogenic, such as systemic lupus erythematosus (100). In IMN, although more than one antigen or antibody has been reported (101, 102), the majority of patients are single-antigen pathogenic. Therefore, the abnormalities of Th cells may not be the main cause of the induction of IMN, and the greater significance of such abnormalities lies in the maintenance of the disease state.

### Th Cells Involved in the Immune Dysregulation of IMN

The differentiation diversity of Th cells is affected by at least two aspects: on the one hand, the differentiation of naive T cells is affected by cytokine signals in the microenvironment; on the other hand, such differentiation is regulated by TCR downstream signals in the cell. Recently, Mikel Ruterbusch et al. proposed a new differentiation model of CD4+T cells *in vivo* (103). The studies on Th cell subsets and related cytokines in IMN were reviewed and recorded in **Table 1**. IMN is identified with obvious Th cells subgroups imbalance, which is mainly reflected in the following aspects:

**Table 1:** Studies of helper T cells in IMN.

Author	Year	Patients	Th cells changes	Related cytokine changes	Reference
Chatenoud L/Cagnoli L/Bannister KM/Rothschild E et al.	1981/1982/1983/1984	12/27/14/8	**Increase:** Ratio of the Th/cytotoxic (OKT4+/OKT8+) or suppressor T cells Decrease: Ratio of the OKT8+T cells	No testing	(104–107)
Zucchelli P et al.	1988	39	**Increase:** Ratio of helper/suppressor T cells(LEU3+/LEU2+)	No testing	(108)
Ozaki T et al.	1992	30	**Increase:** Level of suppressor inducer T (Leu3a+8+) **Decrease:** Level of suppressor T cells (Leu2a+15+)	No testing	(109)
Hirayama K et al.	2002	8	**Increase:** Ratio of Th2 (IL-10+CD4+T cells) **Decrease:** Ratio of Th1 (IL-2+CD4+T cells)	As shown in the left	(110)
Masutani K et al.	2004	24	**Increase:** Ratio of IL-4+Th cells **Decrease:** Ratio of Th1/Th2 (IFN-γ+/IL-4+), positively correlated with urinary protein.	As shown in the left	(111)
Kuroki A et al.	2005	14	**Increase:** Ratio of CD4+T cells, CD4+/CD8+T cells **Decrease:** Ratio of CD8+T cells	**Increase:**IL-10mRNA, IL-13mRNA in PBMC	(84)
Wang B et al.	2011	66	**Increase:** Ratio of CD4+/CD8+ T cells **Decrease:** Number of Treg cells(CD4+CD25+Foxp3+)	No testing	(112)
Shi X et al.	2016	39	**Increase:** Ratio of Tfh cells (CD4+CXCR5+, CD4+CXCR5+PD-1+,CD4+CXCR5+ICOS+, CD4+CXCR5+IL-21+)and ratio of ICOS+/PD-1+Tfh cells	**Increased:** IL-21 in serum	(113)
Michelle Rosenzwajg et al.	2017	25	**Increase:** Frequency of effector memory CD4+T cell **Decrease:** Frequency of Treg (CD25hiCD127lo/-Foxp3+)in CD4+T cells	**Increased:** TNF-α, IL-5, IL-2RA **Decrease:** IL-17, IL-1α, IL-7, and granulocyte-macrophage colony-stimulating factor (GM-CSF) **No change:**IL-35	(83)
Zhang Z et al.	2017	45	**Increase:** Ratio of Tfh cells (CD4+CXCR5+,CD4+CXCR5+ICOS+,CD4+CXCR5+CD154+,CD4+CXCR5+IL-21+,CD4+CXCR5+CD28+), negatively correlated with eGFR and positively correlated with urinary protein.	**Increase:** IL-21, IL-4, IL-10, IL-2, IL-17A, IFN-γin serum Serum IL-21 concentration was negatively correlated with eGFR and positively correlated with urinary protein.	(114)
Cantarelli C et al.	2020	30	**Decrease:** Frequency of Treg (CCR4+CD45RA-CD25+CD127low) **No statistical difference:** Tfh cells, etc	**Increase:** TNF-α in serum **No significant difference:** IFN-γ, IL-4, and IL-17 in CD4+ and CD8+ T cells	(81)
Li, H. et al.	2020	29	**Increase:** Frequency of Th17 (IL-17A+CD4+T),Th2 (IL-4+CD4+T) **Decrease:** Frequency of Th1 (IFN-γ+)and Treg	**Increase:** IL-17A(positive correlation with antibody titer and proteinuria), IL-6, IL-10, IL-13 in serum **No significant difference:** IL-4, IFN-γ, IL-2 in serum	(70)
Cremoni, Marion et al.	2020	56	No testing	**Increase:** IL-17A, IL-4, IL-6 in serum **Decrease:** IFN-γ, IL-10 in serum **No significant difference:** TNF-α, IL-5, IL-13 and GM-CSF	(71)
Roza Motavalli et al.	2021	30	**Increase:** Ratio of Th17/Treg cells **Decrease:** Ratio of Treg (CD4+CD25+CD127−) **No significant difference:** Th17 (CD4+IL-17+)	**Increase:** IL-21, IL-4, IL-10 mRNA in PBMC **Decrease:** expression of FOXP3 in PBMC **No significant difference:** expression of IL-17, IL-23, STAT3, RORγT in PBMC	(72)

First, the CD4+/CD8+T cell ratio increased, and then the Th2/Th1 cell ratio increased, indicating that humoral immunity was dominant in IMN (84). In CD4+T cells, the expression of IL-4 was up-regulated, which was positively correlated with antibody production and disease severity (111). The representative cytokine IFN-γ secreted by Th1 was decreased in IMN (111). Cellular immune-mediated diseases are usually infiltrated by local monocytes and cytotoxic T cells. Although IMN presents as an organ-specific autoimmune disease, there is a local lack of cell infiltration that mediates cellular immunity in the glomerulus, and the generation of proteinuria may be caused by antibody activation of complement that damages the podocytes or antibody affecting podocyte function (115–117). The predictive value of anti-PLA2R antibody titers for clinical prognosis has also been vigorously described (69).

Many studies (112, 115, 118), represented by the rituximab clinical trial conducted by Ronco et al., have shown that IMN reduces Treg cells and destroys immune tolerance, and whether Treg cells can be increased after treatment can predict the therapeutic effect of rituximab. A recent study showed impaired inhibition of Treg cells in IMN (81), which might be attributed to the continuous exposure of antigen and the weakened ability of human immune regulation. TGF-β, IL-35 and IL-10 are the main cytokines secreted by Treg cells that play immunomodulatory roles. Although reductions or no significant changes in TGF-β and IL-35 were observed in IMN, there was an increase in IL-10, and this contradiction could be explained by upregulated regulatory B (Breg) cells in IMN (81). They can also secrete IL-10, but it does not suffice to block the development of the immune response. It was further speculated that the elevated Breg subsets were Br1 cells (119). As mentioned above, there is an antagonistic relationship between Treg and Th17 cells in terms of differentiation, function and other aspects, and imbalance of Th17/Treg has been observed in many autoimmune diseases. Th17 cells in IMN have been a heated topic recently, and studies from different research groups suggested the up-regulation of Th17 and the increase of IL-6 and IL-17A. This indicates that IMN is conducive to Th17 cells differentiation, and also strengthens our confidence that IMN is originated from extrarenal inflammation (115). Increased Th17 cells are also associated with a higher recurrence rate and a higher risk of venous thrombosis (71), which is a concern for clinical treatment.

Autoantibodies are essential to the development and maintenance of IMN, and the production of antibodies requires GC reactions. Tfh cells are professional GC helper B cells, and also serve as the novae of Th cells. In fact, the discovery of Tfh cells has challenged the previous classification of Th cells because their differentiation had been made earlier (103). In addition, it was previously held that the humoral immunity of IMN was driven by IL-4 secreted by Th2 cells, but the present study shows that IL-4 promoting antibody production may come from Tfh cells. Reduction of memory B cells and increase of initial B cells are present in IMN (83), which is consistent with the reports of some other autoimmune diseases (120, 121). An increase in initial B cells, such as Tfh cells, may be associated with the breakdown of tolerance checkpoints (83). The decrease of memory B cells may be caused by the induction of B cells into local tissues by chemokines, or the differentiation into plasma cells to produce antibodies under the action of Tfh cells, or both (85). Two studies from the same group have shown an abnormal increase in Tfh cells in IMN patients, which was correlated with disease severity (113, 114). Earlier studies have also shown that the proportion of CD4+CXCR5+T cells was also up-regulated in the classic model of Heymann nephritis rats (122), a classic animal model of IMN. Nevertheless, there are still many shortcomings, such as discrepancies in the results of studies from the same group. In addition, many questions remain to be explored, such as what causes the abnormal increase in Tfh cells? What is the function of the increased Tfh cells? Are Tfh cells involved in the recurrence of IMN? Research on various lymphocytes in IMN is still insufficient (4), and the role of Tfh cells in the overall immune system of IMN remains to be explored. In addition to the balance between Tfh cells and Treg cells, Tfr cells also form a balance in germinal center. An elevated proportion of circulating Tfh/Tfr cells is found in some autoimmune diseases (123–125), but unfortunately Tfr cells have not been studied in IMN.

We have to point out that although there are many studies on Th cells in IMN, their results are not in good agreement, which is a big obstacle for us to reveal the immunological mechanism of IMN. The underlying reasons may include: A. The included patients are heterogeneous and can be classified by etiological type; B. There are differences in detection methods, especially for cytokines. Measuring cytokine levels after *in vitro* stimulation may differ a lot compared to direct serological tests, because cytokines can be lost in proteinuria; C. The changes of Th cells and cytokines in IMN are small, suggesting that we should include patients with more active immune responses for observation, such as those with higher autoantibody titers. D. All the above studies are based on non-antigen-specific immunity, so the changes of autoreactive T cells may be attributed to a minority of the total T cells that are neglected. Although PLA2R-specific IgG-producing plasma cells have been identified in IMN (126), the changes of self-reactive T cells, such as PLA2R-specific T cells, are unknown in IMN. IMN is an autoimmune disease with antibody response as its core. In this process, abnormal Th cells provide an immune-promoting environment for autoreactive B cells by secreting cytokine. Therefore, the changes in the population and subpopulation of circulating Th cells can still reflect the immunological and pathological state of IMN, but such changes cannot be completely equivalent to those of antigen-specific Th cells. Further studies shall be conducted to clarify this problem, such as the use of flow cytometric analysis or major histocompatibility complex (MHC) tetramer (IST) staining to detect antigen-specific T cells in IMN. Such a study would be beneficial because Treg and Tfh cells do depend on antigen specificity to a certain extent when acting through cellular contact (127), and the study can also provide a basis for specific immunotolerance therapy in IMN. In rheumatoid arthritis, the degree of CD4+ T cell autoreactivity can determine the mode of immune response and influence the treatment prognosis, which is also enlightening for the study of IMN (128, 129).

The local immunological appearance in the kidney is also of concern. The pathological process of IMN is the binding of circulating antibodies to podocyte antigens and the formation of immune complexes deposited on the basement membrane. This process has been widely recognized. In fact, prior to the discovery of autoantigens on podocytes, it was assumed that the antigens of idiopathic membranous nephropathy were located in the tubules under the influence of Heymann nephritis rats, and CD20+B cell infiltration was observed in the tubulointerstitial area of approximately 50% of patients with membranous nephropathy, of which about 50% were focal infiltration (130). This structure is similar to the ectopic lymphoid structure (ELS) but has not been further described. Data from experimental animal models and patients suggest that Tfh cells or cells with Tfh phenotypic characteristics contribute to the maintenance of the structure and function of ELS (131–133). ELS was associated with interstitial inflammation and poor prognosis in IgA nephropathy (134). In autoimmune diseases, Eels or locally infiltrating clusters of B cells often caused harmful effects (135). For example, local autoantibodies were produced in patients with rheumatoid arthritis (136). A recent study by Kyriaki Kolovou et al. has shown that there is localized B cell infiltration in the kidney in renal diseases characterized by podocyte injury, including membranous nephropathy (137). Huimin Li et al. found infiltrating IL-17+ cells in the renal tubule of IMN patients (138). These findings suggest that T-B cell interaction can play a role in the renal tissue of IMN, especially in the renal tubules, and affect the prognosis of the disease, thus providing a new focus for renal pathological diagnosis.

### Th Cells Participating in the Production of IMN Antibodies

In IMN, antibodies against autoantigens are predominantly IgG4, both in renal pathology and in serum, although a small number of other subtypes are also present (139, 140). Why IgG4 is the main pathogenic antibody in membranous nephropathy has always been a problem to be solved. IgG4 is the lowest IgG subtype in the blood of healthy adults, accounting for only 5% (141). Although it has about 90% homology with amino acid sequences of other IgG subtypes, due to changes in individual amino acids, IgG4 is identified with different characteristics, such as Fab arm exchange, weak complement binding force, etc. (142–144). Depending on the environment, IgG4 can play a protective or pathogenic role. In the autoimmune diseases mediated by IgG4, such as IMN and pemphigus, the pathogenic effect of IgG4 is often reflected in blocking the binding of antigen to other proteins and thus affecting its function (90, 144). In IMN, IgG4 combined with THSD7A affects cell adhesion, and thus proteinuria (145), while IgG4 combined with PLA2R may affect IV type collagen fiber adhesion (144, 146), but there are still controversies.

According to V (D) J gene rearrangement, some scholars speculated that IgG4 antibody should be the one with the highest affinity among all IgG subclasses and appear the latest (147). In GC, Tfh cells provide promoting or inhibiting signals to B cells through the competitive mechanism according to their affinity, which is crucial to the production of high-affinity antibodies (148). Among IgG4-mediated autoimmune diseases, including IMN, some other diseases have also demonstrated abnormalities in cTfh cells (83, 84, 110, 113, 114, 149–153), as shown in **Table 2**. These diseases may share some similarities in pathophysiology. Factors that promote the production of IgG4 mainly include two aspects: long-term exposure of allergens or antigens, and the influence of microenvironment created by cytokines, such as IL-4, IL-13, IL-10, IL-21, etc. (90, 154–156). IMN is an autoimmune disease with long-term exposure to autoantigens, and most of the cytokines involved in IgG4 production are abnormal in IMN (see **Table 1**). IL-4 or IL-13 combined with IL-10 can promote antibody conversion to the IgG4 category, while IL-4 combined with IL-21 can stimulate plasma cells to produce IgG4 antibodies (154–156). IL-4 is considered to be the hallmark cytokine of Th2 cells, while IL-21 is believed to be the hallmark cytokine of Tfh cells, even though neither of them serves as the sole source (157, 158). Studies have shown that Tfh cells can also express IL-4 and regulate germinal center response, independent of Th2 cells (159). In fact, Tfh cells may express both IL-21 and IL-4 simultaneously, or in sequence (160). These two cytokines all play a key role in the survival and proliferation of B cells, maturation of antibody affinity and class conversion, and the combined effect of IL-21 and IL-4 can promote the production of IgG4 antibody with the support of CD40 co-stimulatory signal (161), which may be related to the regulation of germinal center response. In addition, IL-21 can promote the production of autoantibodies (158).

**Table 2:** Tfh cells in IgG4-mediated autoimmune diseases.

Disease	Antigen	Target organ	Symptoms	Circulating Tfh	Tfh whether associated with antibodies production	Tfh whether influence the development of disease	Circulating B cells	References
Membranous nephropathy	PLA2R/ THSD7A /others	Kidney(podocytes)	Proteinuria	Both the number and the frequency in Th cells increase	Unclear	Yes, positive	Increase: naive B cells(IgD+CD27-) Decrease: memory B cells(IgD-CD27+,IgD+CD27-)	(83, 84, 113, 114)
Pemphigus	Dsg1/Dsg2	Skin	Flaccid blisters and erosions of the skin and mucous membranes	Frequency in Th cells increases	Yes, positive	Unclear	Unclear	(149, 150)
Myasthenia gravis	MuSK	Muscle/ neuro- muscular junction	Muscle weakness	Frequency in Th cells increases, Tfh17/Tfh1 increases	Yes, positive	Yes, positive	Decrease: B10, CD24+CD38+B cells	(151–153)

### The Role Model of Th Cells in IMN

**Figure 3:**
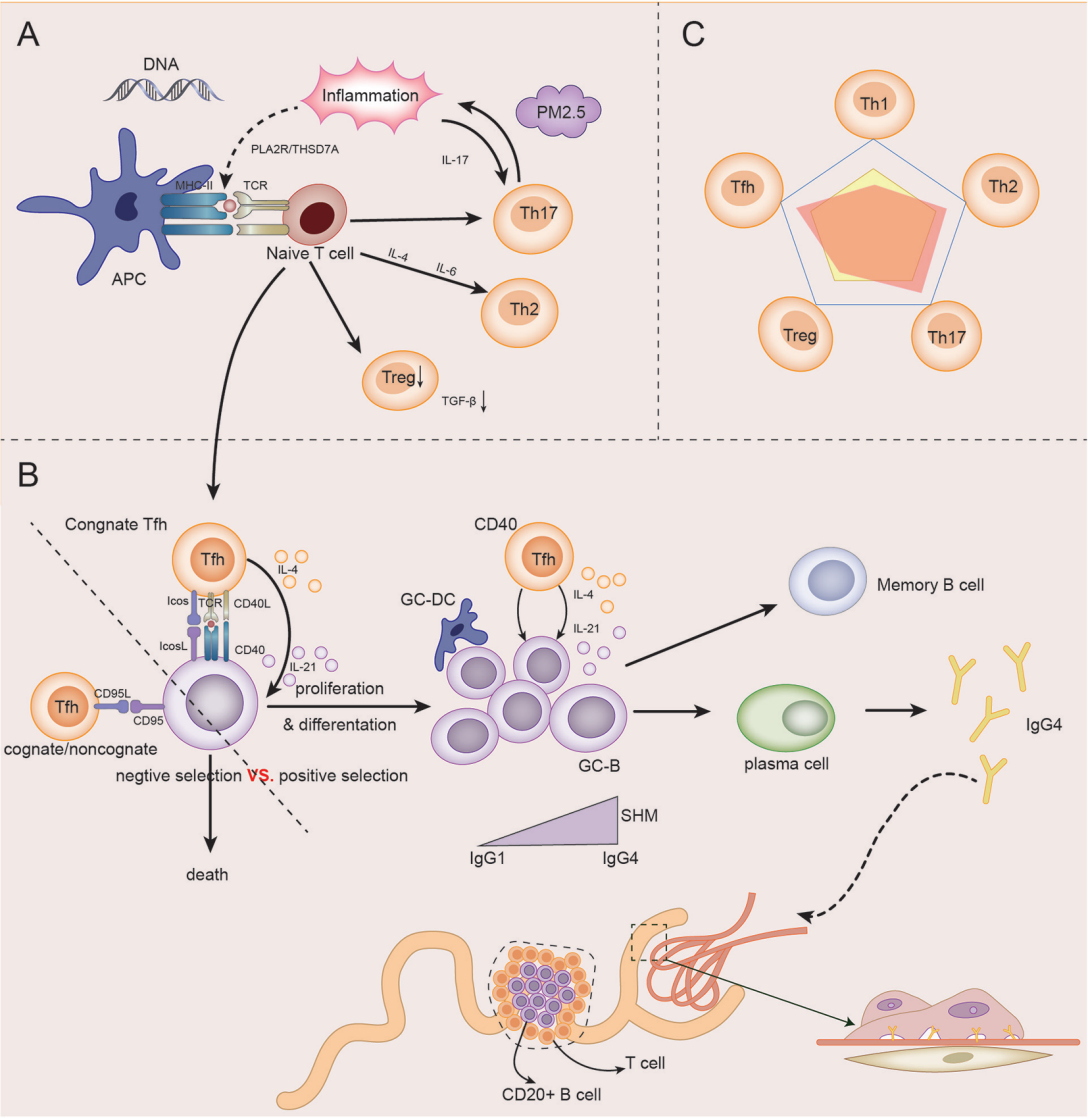
The role model of Th cells in IMN. **(A)** Under the influence of genetic, inflammatory, and environmental factors (PM2.5), antigen-presenting cells (APCs) present their own antigens to juvenile T cells, and then in the initial microenvironment, the immune response develops towards Th2-dominated direction. Infant T cells differentiate into Th17, which in turn participates in and maintains inflammation and promotes immune response. The differentiation of naive T cells to Treg cells decreased, and the immunosuppressive ability decreased. Naive T cells differentiate into Tfh cells and participate in GC reaction. **(B)** In germinal centers, homologous Tfh cells transmit survival signals to B cells *via* CD40L and cytokines (positive selection). Homologous or non-homologous Tfh cells transmit death signals to B cells *via* CD95L (negative selection). The abnormal increase in Tfh cells, which transmit survival signals, gives autoreactive B cells a chance to proliferate and differentiate. Under the action of IL-4 and IL-21 secreted by GC Tfh cells, GC B cells underwent somatic hypermutation (SHM) and antibody affinity maturation. After GC reaction, some B cells become memory B cells and some plasma cells, and begin to secrete IgG4 antibodies. IgG4 circulates to the glomerulus and binds to podocyte antigens (such as PLA2R) to form immune complexes that lead to the pathological appearance of IMN. In addition, under the influence of some factors, T-B cell infiltration may occur in renal tubules, and even form ectopic lymphatic structure, affecting the prognosis of the disease. **(C)** The relationship between the five major Th cell subpopulations in IMN was dominated by Th2, Th17, and Tfh cells, while Treg and Th1 cells were impaired.

Based on the above discussions, genetic, immune, and environmental factors may co-participate in the incidence and development of IMN. In the presence of genetic susceptibility and in extrarenal inflammatory environment, autoantigens represented by PLA2R are presented to T cells. The initial cytokine environment pushes the immune response in a Th2-dominated direction. An abnormal increase in Tfh cells enables the proliferation and differentiation of autoreactive B cells, and assists B cells in completing somatic hypermutation in the germinal center, thus promoting the differentiation of B cells into plasma cells to produce IgG4 antibodies. Inflammation up-regulates Th17 cells and affects autoimmune response and inflammation by secreting cytokines such as IL-17. In addition, Th17 cells, Tfh cells and B cells may be partially liable for the damage of the renal tubulointerstitial region in IMN. The number and function of impaired Treg cells could not be maintained under autoimmune tolerance. The autoimmune response of IMN eventually produces antibodies, which bind to the target antigen on the podocytes, resulting in the classical pathological appearance of IMN, as described in **Figure 3**.

## Treatments for IMN

IMN, as an autoimmune disease, is mainly treated with immunosuppression, which is initiated after a full assessment of the condition, and the patient’s disease status is monitored during the course of treatment. Corticosteroids alone do not work much for the treatment of IMN but are effective when combined with alkylating agents represented by cyclophosphamide (162–164). Cyclophosphamide was originally designed as an antitumor agent and is metabolized by cells to produce phosphoramide mustard (165), which forms cross-links with DNA to achieve cytotoxic effects (166). Cells with high proliferative potential, such as hepatocytes and hematopoietic stem cells, are relatively resistant to cyclophosphamide due to the expression of high levels of aldehyde dehydrogenase (ALDH) (167). Conversely, cyclophosphamide is cytotoxic to mature hematopoietic progenitor cells and almost all lymphocyte subsets (167–169), inducing systemic leukocyte and lymphocyte ablation resulting in rapid suppression of the immune response. However, alkylating agents are associated with a high incidence of adverse events, mainly leukopenia, infection, thrombosis, gonadotoxicity, and increased risk of cancer (170, 171). Calcineurin inhibitors (CNIs) are also widely used in the treatment of IMN, such as tacrolimus and cyclosporine. CNIs can target and block the NFAT signaling pathway, primarily producing an inhibitory effect on T cells, impairing the helper effect of T cells on B cells and thus reducing antibody production. Moreover, some studies have shown that CNIs also have a regulatory effect on the podocyte cytoskeleton (172). The limitations on the clinical use of CNIs lied in their high rate of relapse after drug discontinuation (173) and the association of multiple relapses with progressive renal function (174). Recent studies have shown that the relapse rate after discontinuation of CNIs for IMN can be reduced by the addition of rituximab (175). Rituximab targets the B-cell surface antigen CD20 and cuts the number of B cells other than plasma cells, which can directly reduce antibody titers and induce disease remission (176). In addition to the above drugs, the use of other drugs such as mycophenolate mofetil and belimumab in the treatment of IMN is still being testified.

In addition to remission rates, immunosuppressive therapies for IMN shall also take into account the issues of relapse rates and safety. In terms of the immunological mechanisms of IMN, treatment targeting T or B cells alone may not be comprehensive, and immunosuppressive therapies with multiple targets are yet to be proposed. It has been shown that renal transplant recipient patients treated with a combination of rituximab, tacrolimus, and mycophenolate mofetil are found with Tfh or cTfh cells in the circulation and lymph nodes even when B-cell counts are reduced and GC responses are suppressed (177). Once the B-cell subpopulation recovers after treatment cessation, the residual Tfh may rapidly facilitate B-cell production of auto-reactive antibodies, so the combined or sequential use of rituximab and treatment against Tfh cells may have the potential to reduce relapse rates. Indeed, tacrolimus has a specific inhibitory effect on Tfh cells, which may be due to the greater dependence of Tfh cells on the NFAT signaling pathway (178). In addition, rituximab does not affect increased Th17 cells in IMN, which is associated with relapse and thromboembolism (71). Although many patients can achieve clinical remission with rituximab, maintenance treatment for post-remission immunosuppression, such as targeting Th17 and other Th cells, is also worthy of concern, especially in those patients at high risk of recurrence. It is worth pointing out that the potential therapeutic role of IL-2 in the treatment of autoimmune diseases is gaining increasing attention (179), and recently, a double-blind placebo-controlled trial has demonstrated the efficacy and safety of low-dose IL-2 in the treatment of SLE (180). Different T subpopulations of cells have different affinities with IL-2, with the CD4+FOXP3+ Treg cells subpopulation having a high affinity with IL-2 (181), and Treg cells can be induced to proliferate even at low IL-2, while such dose of IL-2 makes it impossible for other Th cells to proliferate. In addition, IL-2 can inhibit TFH cells responses without relying on Treg cells, which in turn inhibits GC responses and antibodies production (41, 182). By promoting the proliferation of Treg cells and inhibiting the responses of Tfh cells, which are indispensable for the treatment of IMN, IL-2 may have a greater potential in the clinical treatment of IMN.

## Conclusion

IMN is a special autoimmune disease mainly caused by autoantibodies. Although antibodies are secreted by plasma cells, T-B cells also contribute a lot in the immune system, and the imbalance of Th1/Th2, Th17/Treg, Tfh/Tfr cells, and other Th cells subsets in IMN jointly shapes the immunological pathological state of IMN. More studies are needed to fully understand the pathological mechanism of IMN. The application of rituximab shifts the scholars’ attention to the study of B cells in IMN, but Th cells are located in the upstream of B cells, and convincing explanation of the changes in B cell subsets hinges on a good understanding of Th cell subsets, which should also be focused on in clinical treatment.

## Author Contributions

QZ, HD, XL, and BL contributed to the conception and design of the review study. QZ, HJ, ZWL and ZF wrote the first draft of the manuscript. NZ, YG, ZD, XZ, JD and NQZ wrote sections of the manuscript. HR, LY and BL discussed and revised the content of the review article. All authors contributed to the article and approved the submitted version.

## Funding

This work was supported by grants from the National Key Research and Development Project (No. 2019YFC1709402), National Natural Science Foundation of China (No. 81973793 to BL), Capital’s Funds for Health Improvement and Research (No. 2020-2-2234 to BL) and Jiangsu Province TCM science and technology development plan project (No.YB201985).

## Conflict of Interest

The authors declare that the research was conducted in the absence of any commercial or financial relationships that could be construed as a potential conflict of interest.
